# *para tu Salud*: Reduction of Weight and Waistlines by Integrating Exercise Breaks into Workplace Organizational Routine

**Published:** 2007-12-15

**Authors:** Antronette K Yancey, Agustin Lara, Roberto Tapia-Conyer, Pablo Kuri-Morales, Elena Subirats, Yvonne Flores, Ritesh Mistry, William J McCarthy

**Affiliations:** Department of Health Services and Center to Eliminate Health Disparities, Community Research in Cancer Network, Division of Cancer Prevention and Control Research, UCLA Jonsson Comprehensive Cancer Center, Center for Health Policy Research, UCLA School of Public Health; Secretaria de Salud de Mexico (Mexican Ministry of Health), Mexico City, Mexico; Secretaria de Salud de Mexico (Mexican Ministry of Health), Mexico City, Mexico; Secretaria de Salud de Mexico (Mexican Ministry of Health), Mexico City, Mexico; Secretaria de Salud de Mexico (Mexican Ministry of Health), Mexico City, Mexico; Community Research in Cancer Network, Division of Cancer Prevention and Control Research, UCLA Jonsson Comprehensive Cancer Center, Los Angeles, California; Community Research in Cancer Network, Division of Cancer Prevention and Control Research, UCLA Jonsson Comprehensive Cancer Center, Los Angeles, California; Department of Health Services and Center to Eliminate Health Disparities, UCLA School of Public Health, and Community Research in Cancer Network, Division of Cancer Prevention and Control Research, UCLA Jonsson Comprehensive Cancer Center, Los Angeles, California

## Abstract

**Introduction:**

Proactive worksite strategies that change the physical or sociocultural environment(s) to incorporate obligatory physical activity may be necessary to engage sedentary people. This study describes implementation and evaluation of an intervention, *Pausa para tu Salud* (Pause for Your Health), that integrated a brief period of group exercise into the workday.

**Methods:**

An uncontrolled pretest–post-test study design tested the effects of integrating daily 10-minute exercise breaks during paid work time during January 2003 through January 2004. A total of 335 Mexican Ministry of Health office workers provided baseline data as a part of routine annual clinical screening examinations.

**Results:**

Baseline mean body mass index and waist circumferences were 27.8 kg/m^2^ and 87.6 cm for women and 26.6 kg/m^2^ and 89.7 cm for men. Complete data were available for 271 (80.9%) employees at 1-year follow-up. Two-tailed, paired *t*-test comparisons were used. Body mass index decreased by 0.32 kg/m^2^ (*P* = .05), and waist circumference by 1.6 cm (*P* = .0009) overall. The body mass index decrease, however, was significant only for men (−0.43 kg/m^2^, *P* = .03). Multivariate analyses revealed a significant decrease in diastolic blood pressure among women (*z = *−2.04, *P* = .042).

**Conclusion:**

The intervention was associated with significant improvements in both measures of body composition. Substantive health and organizational benefits may result from integrating brief periods of physical activity into the workday if these findings are replicated in randomized controlled trials in other worksites.

## Introduction

Evidence is increasing that low population levels of regular physical activity and high levels of inactivity contribute to the prevalence of chronic disease and obesity (1,2). Higher levels of inactivity in ethnic minority populations than in other populations contribute to obesity and chronic disease disparities in the United States (3). For example, in Los Angeles County, California, the largest and most diverse county in the nation, two in five residents engage in fewer than 10 minutes per week of continuous physical activity (4). In Mexico, inactivity rates of 65% to 80% have been documented among health and social services workers and other urban residents (5,6). Abdominal obesity affects 46.3% of men and 81.4% of women in Mexico (defined there as waist circumference ≥94 cm for men and ≥80 cm for women) (7). Estimates of the prevalence of overweight and obesity combined of 60.7% to 65.3% in Mexico are similar to those in the United States (66.3%) and are rising rapidly (8).

Attention increasingly is turning to upstream public health intervention approaches to reintegrate exercise into daily work and social life. Only about one fifth of the U.S. population embraces active leisure, and little change in participation has accompanied the increases in obesity in recent decades (9). Adults' preference for inactivity may reflect evolutionary programming to conserve energy (10).

Worksite health promotion studies have identified few interventions that include environmental changes to increase exercise. In a recent review of the worksite health promotion literature, Engbers and colleagues (11) identified only three of 13 qualifying randomized controlled trials that focused on encouraging physical activity. Providing exercise space and equipment (on-site fitness facilities), on-site shower facilities, walking tracks, walking route marking, and prompts (signage, riser banners) encouraging stair use were the only strategies employed, with trial quality rated low and only modest self-reported increases in physical activity. Another recent review showed more favorable self-reported physical activity outcomes in studies targeting organizational practices and policies and sociocultural and physical environmental characteristics. Stair prompts, combined with physical improvements to stairwells, have been demonstrated to increase physical activity levels (self-reported and observed) at worksites, at least in the short term; few long-term follow-up data are available (12-14).

However, essentially none of these invitational "pull" strategies incorporate obligatory activity into organizational workday routine (15-17). Pull strategies require voluntary, self-initiated behaviors performed on employee discretionary time, whereas "push" strategies integrate difficult-to-avoid activities in high-exposure settings (e.g., worksites). Pull strategies generally engage only a small and unusually motivated sample of workers who typically are at considerably lower disease risk than the overall employee population. For example, of 3800 employees invited to participate in a recent randomized controlled trial, 244 (6.4%) attended an orientation meeting, 73 of the 240 eligible employees enrolled, and 44 were retained at 24 weeks (18).

Implementation of push strategies that change physical and sociocultural environments to make physical activity essentially obligatory in populous settings may provide substantial opportunity for a large public health impact from a small measured effect. These push strategies include organizational practice and policy changes (e.g., restricting the use of nearby parking to disabled employees). Such physical activity-promoting environments hold promise for engaging even less motivated and more sedentary people (19,20). Employees who engage in pull strategies (e.g., climbing stairs, walking or bicycling to work, using walking tracks or fitness facilities) typically are few and generally younger, healthier, more fit, leaner, and more active than others (20). Sedentary and overweight workers usually have been underrepresented in traditional worksite fitness interventions, thereby limiting potential returns on employer investment in worksite health promotion (21).

Physical activity behaviors might be more amenable than eating behaviors to the cultural influence of the workplace, given the potential for framing activity breaks as an employee benefit (22). Integration of physical activity into workplace routine is particularly important in lower income, Latino, and African American communities, which have more substantial cultural, physical, and economic environmental barriers to physical activity participation (23,24).

Intervention models have emerged that incorporate group physical activity into organizational routine (25-28). Although this early evidence has not yet met the highest standards of methodologic rigor, it is practice-based and translational. This early evidence is precisely what such dissemination-focused frameworks as RE-AIM have identified as necessary for addressing scientific gaps in understanding how to increase the impact of health promotion intervention (29-31). Yancey and colleagues, for example, demonstrated a high level of organizational and individual receptivity to integrating structured 10-minute exercise breaks, called Lift Offs, in meetings, events, and other functions in minority-serving Los Angeles health and social services agencies (20,32,33). The cultural congruence of this approach is evident in ethnic minority communities, in which social participation in spontaneous group physical activity is more common than in mainstream white settings (e.g., middle-aged and older adults dancing to music at parties and holiday celebrations) (23,28). Integrating group physical activity into organizational routine also is consistent with the important role of social support in community settings (34).

We describe here the implementation and evaluation of *Pausa para tu Salud* (Pause for Your Health), an intervention incorporating physical activity into workplace routine similar to the Lift Off exercise breaks. Senior administrators from the Mexican Ministry of Health (MMH) and investigators at the University of California, Los Angeles (UCLA), collaborated on this project. The collaboration resulted from binational project presentations to a meeting of the Board of Directors of the Public Health Institute (of which two of the coauthors [RT, AY] were members) and was conceived when the similarities between *Pausa* and Lift Off were discovered.

## Methods

### Intervention development and formative research

In 2001, the National Center for Epidemiological Surveillance and Disease Prevention (CNVE) of the MMH evaluated the health status of a sample of employees. Fifty percent of those surveyed had one or more risk factors for cardiovascular disease, primarily overweight or obesity. *Pausa para tu Salud*, created by CVNE staff, was a subset of a larger populationwide intervention program to promote physical activity called *Por tu Salud, Actívate* (For Your Health, Move), launched in 2003. The objectives of the intervention were to 1) promote more active lifestyles conducive to preventing and controlling chronic disease and to enhancing overall employee health and well-being, 2) introduce the habit of routine nondiscretionary-time physical activity into the workplace, and 3) foster healthier interpersonal relationships at work. As with Lift Off, social cognitive theory and social ecologic models provided the basis for development of the *Pausa* intervention.

*Pausa para tu Salud* began in January 2003. The MMH Director of Health Promotion and Disease Prevention (coauthor RT) strongly encouraged all office employees to participate in the exercise breaks (*pausas*) as a regular work activity. *Pausas* were conducted at a specific time each morning (11–11:30 AM), about halfway through most employees' workdays. They began as 10 minutes of light stretching and dance movements and gradually increased in intensity as participants' fitness levels improved. Each day, after two broadcast reminder announcements, music for the *Pausa* was broadcast over the intercom system in the main administration building. Music selections varied frequently in response to employee suggestions. The *Pausa* routines also were varied to expose employees to different types of strength, flexibility, and aerobic conditioning exercises. Employees uncomfortable exercising with the group were supervised while they participated privately at their workstations. Other project intervention activities included posting of stair prompts, distribution of written materials, and encouragement of staff by their supervisors and MMH leadership to engage in additional physical activity outside the workplace.

### Sample

A total of 335 MMH health and social services office workers provided baseline data in January 2003 and 1-year follow-up data in January 2004 as part of routine annual clinical screening examinations conducted on all staff by MMH professionally trained medical personnel. Clinic staff collected sociodemographic (age, sex, job location), anthropometric (waist circumference, weight, height), and physiologic (blood pressure) data according to standard protocols. All employees were potentially included; the evaluators had no basis for excluding employees for medical reasons because the secondary data available to them included no information about concurrent medical conditions.

### Measures

**Sociodemographic**

Clinic staff recorded each employee's age, sex, and job location at baseline. No effort was made to track any changes in job location.

**Anthropometric**

For all anthropometric measures, the employee disrobed and was measured in undergarments only. Waist circumference was measured to the closest .5 cm using a standard plastic tape measure at the minimum circumference around the waist or just above the iliac crest for those with no minimum circumference around the waist. Body weight was measured in kilograms with a balance beam scale. No attempt was made to control for time of day. Height was measured to the closest ½ cm with a wall-mounted stadiometer with the employee in the recommended Frankfort plane stance during the measurement (i.e., back of the heel resting against the wall). From the height and weight measurements, body mass index (BMI) was calculated.

**Physiologic**

After a 5-minute rest in a sitting position, the employee's blood pressure was measured to the closest 2 mm of mercury with a mercury sphygmomanometer, with the employee's arm resting on a table surface approximately 5 cm above waist level. No fitness measures were collected during employees' annual medical examination.

### Data management and analysis

We used an uncontrolled, pretest–post-test study design. In accordance with institutional review board requirements, baseline and 1-year follow-up data without personal identifiers were provided to UCLA research staff. We analyzed the data using Stata version 10 (35). To accommodate the repeated measures data, the data were modeled using STATA's mixed effects regression procedure (xtmixed). To compare the data with past research, we also reported means and *t*-test results.

## Results

We matched baseline and follow-up data through an iterative and systematic approach using the initial order of these data, aided by triangulation of study (worksite designation) and demographic (e.g., sex) characteristics. At baseline, the 335 participants represented approximately 90% of all employees working in the MMH main administration building, excluding only those whose leave (medical, vacation) or work-related travel prevented their routine annual physical examinations. Sixty-two percent of participants were women. Mean age of participants was 48.9 years (standard deviation = 16.8; range: 18–87 years). Mean BMI and waist circumference were 27.8 kg/m^2^ and 87.6 cm for women and 26.6 kg/m^2^ and 89.7 cm for men (Table 1).

Sixty-four employees lost to follow-up after baseline analyses were dropped from the study. The most common reasons for loss to follow-up were leave or work-related travel, with minor attrition because of job change or retirement. Independent group *t*-test results indicated no baseline differences between the 271 respondents retained and the 64 lost to follow-up (*t* [range] = 1.2–2.0, *P* > .12–.48) (Table 2). The remaining 271 participants (80.9% of all employees screened at baseline as eligible for participation) were retained for post-test analyses. All regression analyses included age as a covariate. Subgroup analyses were stratified by sex.

Although we observed an intervention effect for body composition, measured either as weight, BMI, or waist circumference, results varied by sex. Overall, weight decreased by 1.01 kg (*z *= −2.08, *P* = .038); BMI decreased by 0.32 kg/m^2^ (*z *= −1.99, *P* = .047); and waist circumference decreased by 1.6 cm (*z *= −3.56, *P* < .0005). Weight decreased for men (*z *= −2.22, *P* = .026) but not for women; BMI decreased significantly for men (*z *= −2.35, *P* = .019) but not for women (mean: −0.43 kg/m^2^, *P* = .03 for men vs −0.25 kg/m^2^, *P* = .28 for women). Waist circumference decreased significantly for both men (mean: 1.9 cm, *z *= −4.06, *P* < .0005) and women (mean: 1.4 cm, *z *= −2.12, *P* = .034).

Using mixed effects model regression that included the influence of changes in BMI, waist circumference, and age, we evaluated changes in systolic and diastolic blood pressure (an unobtrusive marker for cerebrovascular health) over the 1-year intervention period. The change in systolic blood pressure was not significant and was significantly related only to age (overall *z *= 3.03, *P* = .002). The 1-year decrease in diastolic blood pressure, however, was significant for women (*z *= −2.04, *P* = .042), was significantly related to BMI decrease (overall *z *= 2.93, *P* = .003), and was significantly related to age for both sexes (overall *z *= 5.27, *P* < .0005).

## Discussion

This project demonstrated an association between exposure to the *Pausa* intervention and a significant decline in all three measures of body composition, although subgroup analyses indicated less consistent decreases for women than for men. Furthermore, the change in body composition, as measured by BMI, was associated with a significant decline in diastolic blood pressure among women. These results are all the more impressive considering that the secular trend in this and in most adult populations is for increasing excess body fat with each cohort and increasing prevalence of obesity and hypertension with age (36).

*Pausa* findings are consistent with published evidence of salutary psychological, behavioral, clinical, and organizational outcomes resulting from brief periods of physical activity (20,24-27,33). However, because no information was collected during the routine medical examination about employees' current physical activity levels, we could not determine whether improvements in body composition and diastolic blood pressure can be attributed exclusively to the 10 minutes of daily calisthenics. The 10-minute worksite exposure to moderate physical activity might have heightened awareness of participants' physical fitness levels (33) and consequently might have increased motivation to exercise outside of the worksite (37). The intervention effects demonstrated here probably are attributable to the employees' brief physical activity periods, either alone or synergistically with other intervention elements.

These data suggest the use of brief periods of group physical activity during paid time in the workplace to address the risk for chronic disease and obesity in diverse urban U.S. communities. The findings are consistent with the estimate by Hill and colleagues (38) of an energy gap as small as 100 kcal/day accounting for the weight gain of the vast majority of the population. If these preliminary findings from the *Pausa* intervention are replicated in workplace randomized controlled trials, then we can be more confident of the potential for substantive health, organizational, and societal benefits from integrating brief periods of physical activity into workplace routine.

Groups likely to benefit most from this approach are the more sedentary and overweight population subgroups, many of which are ethnic minorities or women. These subgroups historically have shown little interest in traditional workplace physical activity promotion efforts, perhaps because such efforts usually were competitive, leisure-time, individual, or sports-oriented physical activity promotion efforts. Collectively engaging workers in exercising to musical accompaniment is consistent with women's physical activity preferences in general and is culturally grounded in the traditions, norms, and values of many ethnic minorities (19,23,28). Diffusing such culturally salient innovations (i.e., those arising within the context of the culture of origin of the minority group targeted) may be more feasible and effective than efforts to adapt, implement, and disseminate interventions originally developed in mainstream research settings with predominantly affluent, white populations (23,28).

The *Pausa* study has several limitations. First, our study group lacked a control group; however, researchers repeatedly have confirmed the existence of a secular trend for rising BMI with time and with increasing age (36). The downward trend in body composition documented here is inconsistent with this secular trend. In addition, selection bias toward healthier workers might have contributed to the healthful changes because data were not available to compare study participants with the small group of workers not presenting for their annual clinical examinations. Other limitations include the absence of information about exposure dose, precluding discernment of dose-response relations between intervention participation and outcomes; lack of survey self-report data to ascertain whether changes were attributable to workplace physical activity, extramural physical activity, dietary changes, or some additive or synergistic combination of these; and inability to isolate intervention components' effects. However, physician risk-reduction counseling produces only modest long-term effects on physical activity and none consistently on BMI (39). Similarly, stair prompts have produced only short-term effects on physical activity (40).

The increasing cultural diversity of the U.S. working population includes many Latinos. This fact, combined with the inactivity of jobs in the increasingly knowledge-driven and information technology-laden postmodern economies of developed nations makes incorporation of brief periods of activity a promising, practical intervention. Physical activity behaviors may be even more amenable to the microcultural influence of the workplace than eating behaviors because interventions focusing on physical activity are less controversial than those focusing on dieting and reducing excess weight. Physical activity also might stimulate positive dietary changes by, for example, increasing preference for less highly sweetened beverages and for more water-bearing foods (41). Sedentary workers typically have not been involved in traditional worksite fitness efforts, limiting the return on employer investment. Success in engaging them in everyday moderate physical activity, however, is critical to the accrual of greater and more rapid benefits of participation in health promotion programs by workers and employers.

## Figures and Tables

**Table 1:** Descriptive Statistics for Baseline and 1-Year Follow-Up Anthropometric and Blood Pressure Measures of Employees (N = 335) Participating in the *Pausa para tu Salud* Intervention

		Anthropometric Measure							Blood Pressure			

		Body Mass Index (kg/m^2^)		Height (m)	Weight (kg)		Waist Circumference (cm)		Systolic (mm Hg)		Diastolic (mm Hg)	
Assessment		1	2	1	1	2	1	2	1	2	1	2
Women	Mean	27.76	27.50	1.55	66.88	65.64	87.58	86.25	121.51	120.51	77.56	77.09
	SE	0.46	0.44	0.005	1.02	1.08	0.98	0.69	0.92	0.85	0.64	0.66
	n	170	160	170	207	170	207	170	207	170	207	170
Men	Mean	26.65	26.14	1.69	76.06	74.41	93.06	90.47	122.08	122.16	78.01	78.71
	SE	0.48	0.49	0.009	1.28	1.30	1.19	1.23	1.02	0.89	0.70	0.65
	n	110	99	110	128	101	128	101	128	101	128	101
Total	Mean	27.32	26.98	1.61	70.39	69.10	89.67	87.82	121.73	121.12	77.73	77.69
	SE	0.34	0.33	0.006	0.84	0.87	0.77	0.77	0.69	0.63	0.48	0.48
	n	280	259	280	332	271	332	271	332	271	332	271

1 indicates baseline; 2 indicates 1-year follow-up; SE indicates standard error.

**Table 2:** Comparisons of Baseline and 1-Year Follow-Up Anthropometric and Blood Pressure Measures by Paired *t* Test, *Pausa para tu Salud* Intervention

Variable	Sample	No. Participants	Mean Difference	SE	Paired *t* Test	95% Confidence Interval	*P* Value
**Weight**	Total	271	1.01	0.45	2.22	0.11 to 1.90	.03
	Women	170	0.85	0.64	1.32	−0.42 to 2.11	.19
	Men	101	1.28	0.56	2.26	0.16 to 2.40	.03
**Body mass index^a^**	Total	259	0.32	0.16	2.00	0.01 to 0.63	.05
	Women	160	0.25	0.23	1.08	−0.21 to 0.71	.28
	Men	99	0.43	0.19	2.32	0.43 to 0.80	.03
**Waist circumference**	Total	271	1.59	0.47	3.35	0.66 to 2.52	.0009
	Women	170	1.41	0.69	2.02	0.04 to 2.78	.05
	Men	101	1.90	0.51	3.76	0.90 to 2.91	.0003
**Systolic blood pressure**	Total	271	0.69	0.51	1.35	−0.31 to 1.69	.18
	Women	170	1.15	0.71	1.61	−0.26 to 2.55	.71
	Men	101	−0.08	0.66	−0.12	−1.38 to 1.22	.90
**Diastolic blood pressure**	Total	271	0.40	0.39	1.03	−0.36 to 1.16	.30
	Women	170	0.65	0.56	1.16	−0.46 to 1.75	.25
	Men	101	−0.02	0.43	−0.05	−0.88 to 0.84	.96

SE indicates standard error.

a Sample size was lower for BMI because height data were missing for 12 participants.
